# Patient pathway analysis of rifampicin-resistant TB diagnostic and treatment delays

**DOI:** 10.5588/ijtldopen.24.0469

**Published:** 2025-02-01

**Authors:** S. Ge, Z. Feng, L. Lin, R. Li, Y. Zhang, L. Song, A. Wang, Y. Lan, Y. Li, Z. Huang, C. Cai, X. Wang, Q. Ruan, H. Yu, M. Tang, H. Yi, Y. Chen, F. Sun, Y. Li, W. Zhang

**Affiliations:** ^1^ Department of Infectious Diseases, Shanghai Key Laboratory of Infectious Diseases and Biosafety Emergency Response, National Medical Center for Infectious Diseases, Huashan Hospital, Fudan University, Shanghai, China;; ^2^ Department of Tuberculosis, The Third People's Hospital of Bijie, Bijie, China;; ^3^ Department of Pneumology and Critical Care Medicine Respiratory Division II, Affiliated Hospital of Zunyi Medical University, Zunyi, China;; ^4^ Department of Tuberculosis, The Third People's Hospital of Liupanshui, Liupanshui, China;; ^5^ Department of Tuberculosis, Guiyang Public Health Clinical Centre, Guiyang, China;; ^6^ Department of Infectious Diseases, The People's Hospital of Anshun, Anshun, China;; ^7^ Department of Infectious Diseases, The First People's Hospital of Huaihua, Huaihua, China;; ^8^ Department of MDR-TB, Hunan Province Chest Hospital, Changsha, China;; ^9^ Department of Tuberculosis, Hangzhou Red Cross Hospital, Hangzhou, China;; ^10^ Shanghai Sci-Tech Inno Center for Infection & Immunity, Shanghai, China.

**Keywords:** diagnostic delay, treatment delay, drug susceptibility testing, rapid molecular testing

## Abstract

**BACKGROUND:**

Barriers to the diagnosis and treatment of rifampicin-resistant TB (RR-TB) have not been fully elucidated. This study aimed to map the diagnostic and treatment delays among patients with RR-TB in China and investigate related factors.

**METHODS:**

Between June and July 2023, the diagnostic and treatment pathways of patients with RR-TB were obtained through interviews at eight hospitals across China. Information on the TB service of hospitals was collected via telephone.

**RESULTS:**

Ninety-eight patients were included. On average, each patient required 4.6 visits to start RR-TB treatment. The median delay from illness onset to RR-TB treatment was 238.0 days (IQR 85.8–469.2), primarily driven by the delay between TB diagnosis and identifying rifampicin (RIF) resistance (median: 43.5 days, IQR 3.0–160.5). Referral to appropriate hospitals (adjusted hazard ratio [aHR] 2.32; 95% CI 1.37–3.92) or considering drug susceptibility testing (DST) when initiating treatment (aHR 2.56, 95% CI 1.39–4.72) significantly reduced delay between TB diagnosis and identifying RIF resistance, while stigma regarding TB (aHR 0.61, 95% CI 0.38–0.98) was an independent risk factor.

**CONCLUSIONS:**

Patients with RR-TB experienced substantial delays in identifying RIF resistance. Referring patients to hospitals with molecular DST capabilities and awareness may reduce these delays.

TB remains one of the leading causes of morbidity and mortality worldwide, with the WHO estimating that 10.8 million people fell ill with TB and 1.25 million died from the disease in 2023.^1^ The emergence and rapid transmission of drug resistance pose a growing challenge to TB control efforts.

Optimising the diagnosis and treatment process of patients with TB is crucial for TB control. Long delays lead to poor outcomes for individuals,^2^ and contribute to ongoing transmission. Understanding the current status of patients’ diagnostic and treatment processes is essential for optimisation.

Previous studies have discussed patients’ diagnostic and treatment delays and provided valuable evidence.^3–7^ However, studies specifically addressing rifampicin-resistant TB (RR-TB) have been relatively limited. Existing research on RR-TB often focuses on specific parts of diagnostic and treatment delay, patients diagnosed via a rapid diagnostic method,^2,8–11^ or surveillance data for macroscopic descriptions.^12,13^ The number of studies analysing the diagnostic and treatment delays of RR-TB from a patient-centred perspective is limited.

This study aimed to address this gap by collecting comprehensive information on patients’ entire diagnostic and treatment process with RR-TB through interviews conducted in China. The study mapped the diagnostic and treatment pathway of patients with RR-TB in China, described the diagnostic and treatment delays of RR-TB, and investigated related factors.

## METHODS

### Study design

Between June 2023 and July 2023, a multicentre, retrospective observational study was conducted across eight TB-designated hospitals encompassing the western, central, and eastern China regions. The participating hospitals included the Affiliated Hospital of Zunyi Medical University, the Third People’s Hospital of Bijie, the Third People’s Hospital of Liupanshui, Guiyang Public Health Clinical Centre, the People’s Hospital of Anshun, the First People’s Hospital of Huaihua, Hunan Province Chest Hospital, and Hangzhou Red Cross Hospital. The inclusion criteria were patients aged ≥18 years with pulmonary RR-TB confirmed by molecular or phenotypic drug susceptibility testing (DST). Each hospital was visited for 3 days. All patients visiting the hospitals during the study period, including hospitalised and outpatient patients, who fulfilled the inclusion criteria during the visit were invited to participate, and those who consented were included.

Data were collected through interviews and medical records, focusing on sociodemographic characteristics, clinical presentations, and the care-seeking pathway. The care-seeking pathway included the date of illness onset and medical consultations, names of hospitals, reasons for choosing the hospitals, travel time from home to those hospitals, laboratory and radiological tests performed and their findings, and the diagnoses and medical advice.

Following the interviews, information on hospital levels and TB diagnostic services, including radiography, *Mycobacterium tuberculosis* culture, acid-fast bacilli smear and DST were collected through telephone consultations with the hospitals.

The study was approved by the Ethics Review Committee of Huashan Hospital, Fudan University, Shanghai, China. Informed consent was obtained from all participants in the study.

### Definitions

Four key steps were identified in the patient pathway: the first medical visit (Step 1), diagnosis of TB (Step 2), diagnosis of RR-TB (Step 3), and initiation of treatment for RR-TB (Step 4). Four corresponding delays were defined as follows:

*Patient delay:* The time interval from symptom onset (or radiographic abnormalities) to the first medical visit.
*TB diagnostic delay:* The time interval from the first medical visit to TB diagnosis.
*Rifampicin (RIF) resistance diagnostic delay:* The time interval from TB diagnosis to the identification of RIF resistance (diagnosis of RR-TB).
*Treatment delay:* The time interval from the RR-TB diagnosis to the initiation of treatment for RR-TB.

Smoking history was defined as having smoked ≥100 cigarettes in a lifetime and/or currently smoking. Alcohol consumption was defined according to the guidelines of the National Institute on Alcohol Abuse and Alcoholism (Bethesda, MD, USA). Hospitals were classified based on the three-tier hospital system in China: level 0, level 1, level 2, and level 3 (Supplementary Table S1).^14^

### Statistical analysis

Categorical variables were described with counts (percentages or proportions), and continuous variables were described with means (standard deviation [SD]) or medians (interquartile range [IQR]). Univariate and multivariate Cox proportional hazards regression models were used to identify factors associated with RR-TB diagnosis and treatment initiation. Hazard ratios (HRs), adjusted hazard ratios (aHRs) and their corresponding 95% confidence intervals (CIs) were reported. One-way analysis of variance, Kruskal-Wallis test, χ^2^ test and Fisher’s exact test were used to compare continuous variables, as appropriate. All analyses were conducted in R v4.3.1 (R Computing, Vienna, Austria). *P* < 0.05 were considered statistically significant.

## RESULTS

### Baseline characteristics of the participants

A total of 98 bacteriologically confirmed pulmonary RR-TB patients were included in this study. The baseline characteristics are shown in Table 1. The median age was 42.5 years (IQR 28.0–55.8), and 34 (34.7%) were female. The median annual household income per capita was USD3,288. Thirty-five (35.7%) patients had a history of TB. The most common symptoms at onset were any form of cough (69.4%). During this clinical course, 68 (69.4%) patients received treatment for drug-susceptible TB (DS-TB). Patient characteristics stratified by hospital are given in Supplementary Table S2.

**Table 1.** Baseline characteristics of study population.

Variables	Participants (*n* = 98) *n* (%)
Age, years, median [IQR]	42.5 [28.0–55.8]
Female sex	34 (34.7)
Living in rural areas	56 (57.1)
Annual household income per capita, USD, median [IQR]	3,288 [1,370–5,479]
Smoking history	40 (40.8)
Alcohol consumption	13 (13.3)
Concomitant condition	
Previous history of TB	35 (35.7)
Diabetes mellitus	17 (17.3)
BMI <18.5 kg/m^2^	28 (28.6)
Onset of illness	
Any cough	68 (69.4)
Sputum	46 (46.9)
Weak	39 (39.8)
Fever	32 (32.7)
Prolonged cough (≥2 weeks)	27 (27.6)
Radiographic abnormalities	14 (14.3)
Had received first-line TB treatment	68 (69.4)
Duration of symptoms at presentation, days, median [IQR]	30.0 [11.0–160.0]

IQR = interquartile range; USD = US dollar; BMI = body mass index.

### Patient pathway description

RR-TB treatment initiation dates were recorded for 98.0% (96/98) participants since two participants had not yet initiated RR-TB treatment when surveyed and were unreachable after the survey. The 96 participants experienced 446 medical visits, averaging 4.6 visits per patient. The median interval from illness onset to RR-TB treatment initiation was 238.0 days (IQR 85.8–469.2). From the first medical visit to RR-TB diagnosis, participants experienced 368 visits, with an average of 3.8 visits per patient; 50.0% (49/98) required three or more visits to be diagnosed with RR-TB.

Participants reported 130 hospitals visited, of which we contacted 95.4% (124/130) by phone to collect diagnostic service information. For the first visit, the coverage and usage rates of radiography service were over 90%, higher than those of *Mycobacterium tuberculosis* culture and acid-fast bacilli smear (Supplementary Table S3). In the second visit, the coverage rates increased to over 90% for all three tests.

For the first visit, 40.7% of hospitals could provide phenotypic DST, while only 13.5% of patients used it. Molecular DST was available at 40.2% of hospitals, and the usage percentage was 48.6% (Supplementary Table S3). For all patients receiving molecular DST, this was performed directly on sputum samples rather than on culture isolates. Only 20.4% of patients were diagnosed with RR-TB at the first visit. The coverage rate of phenotypic DST and molecular DST increased during later visits, but the usage percentage remained relatively stable. Information about hospital level, selection and distance related to the latter four key steps is shown in Supplementary Figure S1.

### Diagnostic and treatment delays in all participants

The median patient delay, TB diagnostic delay, RR-TB diagnostic delay and treatment delay were respectively 24.5 days (IQR 2.2–123.5), 2.5 days (IQR 0.0–14.8), 43.5 days (IQR 3.0–160.5) and 10 days (IQR 4.0–30.2) (Figure). Among these delays, the RR-TB diagnostic delay was the longest. Over half (55.1%) of the participants spent more than 28 days from TB diagnosis to RR-TB diagnosis. Treatment delay exceeded 28 days for 26.0% of participants.

**Figure.**
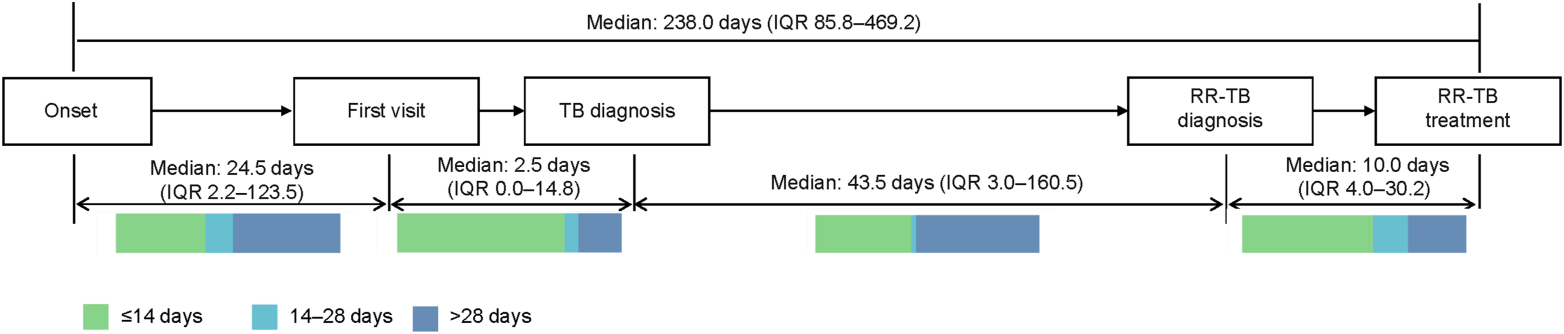
Diagnostic and treatment delays experienced by participants, expressed in median [IQR] number of days. Bars below the four types of delays show the distribution of time intervals for all participants. IQR = interquartile range; RR-TB = rifampicin-resistant TB.

### Modes of RR-TB diagnostic delay

Further analysis of the pathway from TB diagnosis to RR-TB diagnosis identified three diagnostic modes in 91.8% (90/98) of participants. In Mode 1, 31 participants were diagnosed with TB and promptly initiated DS-TB treatment regimens without concern for drug resistance. These patients were eventually diagnosed with RR-TB after a prolonged period on DS-TB treatment. In Mode 2, 30 participants began the treatment for DS-TB upon TB diagnosis, with DST performed subsequently. In Mode 3, 29 participants underwent molecular DST at the time of TB diagnosis. Information on the remaining eight participants is detailed in Supplementary Table S4.

The patient and TB diagnostic delays were similar across the three modes (Table 2). Those who initiated DS-TB regimens without concern for drug resistance experienced the longest RR diagnostic delay (182.0 days, IQR 82.0–281.0), while those who received molecular DST at TB diagnosis had negligible RR-TB diagnostic delay (0.0 days, IQR 0.0–4.0). Those who initiated the DS-TB regimen with ongoing DST had an RR-TB diagnostic delay of 32.5 days (IQR 3.0–68.8).

**Table 2.** Diagnostic and treatment delay characteristics by delay mode.^*^

	Overall (*n* = 90)	Mode 1 (*n* = 31)	Mode 2 (*n* = 30)	Mode 3 (*n* = 29)	*P*-value
Delay, days, median [IQR]					
From onset to first visit	26.0 [2.3–123.5]	22.0 [7.5–91.5]	38.5 [11.8–177.5]	19.0 [0.0–99.0]	0.554
From first visit to TB diagnosis	2.5 [0.0–14.8]	2.0 [0.0–16.5]	1.5 [0.0–6.3]	4.0 [1.0–15.0]	0.326
From TB diagnosis to RR-TB diagnosis	32.5 [2.0–128.8]	182.0 [82.0–281.0]	32.5 [3.0–68.8]	0.0 [0.0–4.0]	<0.001
From RR-TB diagnosis to RR-TB treatment	10.0 [4.0–27.8]	6.0 [2.5–16.5]	21.0 [8.0–58.0]	10.0 [4.0–21.3]	0.015
From TB diagnosis to DS-TB treatment	0.0 [0.0–1.3]	0.0 [0.0–1.0]	0.0 [0.0–3.0]	—	—
Duration of treatment for DS-TB, days, median [IQR]	90.0 [35.0–247.0]	167.0 [81.5–268.0]	43.5 [9.8–122.3]	—	—

* Mode 1 = those who initiated DS-TB treatment without concern for drug resistance; Mode 2 = those who initiated DS-TB treatment with ongoing DST; Mode 3 = those who received molecular DST at TB diagnosis.

IQR = interquartile range; RR-TB = rifampicin-resistant TB; DS-TB = drug-susceptible TB.

### Factors associated with RR-TB diagnostic delay

As previously stated, the delay was principally driven by the long duration from TB diagnosis to RR-TB diagnosis. Therefore, we further analysed the potential risk factors of RR-TB diagnostic delay. Univariate analysis suggested that having stigma regarding TB, receiving treatment for DS-TB before RR-TB diagnosis, being referred to appropriate hospitals by physicians at TB diagnosis, visiting more hospitals before RR-TB diagnosis, and different delay modes were related to RR diagnostic delay (Supplementary Table S5, Table 3). Multivariate analysis showed that those who were referred to appropriate hospitals (aHR 2.32, 95% CI 1.37–3.92) or initiated a DS-TB regimen with ongoing DST (aHR 2.56, 95% CI 1.39–4.72) had significantly reduced RR diagnostic delay. Having stigma regarding TB (aHR 0.61, 95% CI 0.38–0.98) was significantly associated with longer RR-TB diagnostic delay.

**Table 3.** Factors associated with faster RR-TB diagnosis.

	Univariable Cox regression		Multivariable Cox regression	
	HR (95% CI)	*P*-value	Adjusted HR (95% CI)	*P*-value
High school degree or above	1.43 (0.95–2.16)	0.087	1.19 (0.73–1.94)	0.489
Had prolonged cough (≥2 weeks) before the first visit	0.66 (0.42–1.04)	0.076	0.72 (0.43–1.19)	0.198
Experienced TB stigma	0.52 (0.34–0.80)	0.002	0.61 (0.38–0.98)	0.040
Received DS-TB regimens before RR-TB diagnosis	0.07 (0.04–0.15)	<0.001	0.22 (0.02–2.19)	0.198
Proper hospitals were recommended at TB diagnosis	1.70 (1.12–2.60)	0.014	2.32 (1.37–3.92)	0.002
Molecular DST available at TB diagnosis	1.52 (1.00–2.32)	0.052	1.55 (0.90–2.67)	0.116
Number of hospitals visited before RR-TB diagnosis	0.68 (0.52–0.88)	0.003	0.95 (0.71–1.26)	0.715
Delay modes^*^				
Mode 1	Reference		Reference	
Mode 2	2.89 (1.70–4.90)	<0.001	2.56 (1.39–4.72)	0.003
Mode 3	27.01 (11.95–61.05)	<0.001	5.31 (0.52–54.1)	0.159
Others	0.64 (0.28–1.46)	0.288	0.71 (0.28–1.76)	0.458

* Mode 1 = those who initiated DS-TB treatment without concern for drug resistance; Mode 2 = those who initiated DS-TB treatment with ongoing DST; Mode 3 = those who received molecular DST at TB diagnosis.

RR-TB = rifampicin-resistant TB; HR = hazard ratio; CI = confidence interval; DS-TB = drug-susceptible tuberculosis; DST = drug susceptibility testing.

### Factors associated with treatment initiation

RR-TB treatment delay was the second longest, and the associated factors were also investigated. Univariate analysis found that the first DST method, age, smoking, having sputum before the first visit and visiting more hospitals before RR-TB diagnosis was related to RR-TB treatment delay (Supplementary Tables S5 and S6). Multivariate analysis showed that those with older age (aHR 1.03, 95% CI 1.01–1.04) and were diagnosed by molecular DST (aHR 2.87, 95% CI 1.45–5.65) were more likely to initiate RR-TB regimens in time, while those who received DS-TB treatment with ongoing DST were less likely to initiate RR-TB regimens in time (Supplementary Table S6).

## DISCUSSION

This patient-pathway analysis described the diagnostic and treatment process in detail and determined the gaps in the RR-TB healthcare cascade in China, revealing an unacceptable delay in identifying RIF resistance. These delays contribute to suboptimal treatment and ongoing transmission, underscoring the need for more timely assessments of drug resistance. This study provided important evidence for subsequent optimisation of TB control strategies.

We found that most patients initially sought care at nearby hospitals for convenience, although less than half of these facilities had the capacity to diagnose RR-TB. Only 20.4% of patients were successfully diagnosed with RR-TB at the first visit. Even in subsequent visits, the DST coverage and percentage of actual tests performed were inadequate. Consequently, patients had to visit other, more distant, higher-level with better TB diagnosis and treatment capabilities. On average, each patient needed to visit 4.6 times to initiate RR-TB treatment. These results indicated a mismatch between hospital choice and TB medical resources. This also indicated that effective referrals might play an important role in improving diagnosis and treatment.

The study revealed a median diagnostic and treatment delay of 238 days for RR-TB patients, which took far longer than that of patients with the overall TB population.^15–17^ Prolonged delay indicated that its associated transmission could also be more widespread. Previous studies have identified the transmission of multidrug-resistant TB (MDR-TB) as a primary cause of the high burden of DR-TB,^18^ and our findings were consistent with this, as they indicated the prolonged period during which RR-TB patients remained infectious. Also, 64% of patients in this study had no TB history, and 56% of patients received less than 2 months of anti-TB treatment before diagnosis, indicating a potentially substantial proportion of patients with primary DR-TB.

With advances in TB diagnostic technology and medical procedures, the delay in TB diagnosis has significantly improved. Many of the hospitals in this study overlapped with those in the patient pathway analysis we conducted in 2019 for DS-TB patients.^19^ However, we have observed a significant reduction in the delay of TB diagnosis, from 20 days down to 2.5 days. This improvement may be attributed to patients quickly visiting hospitals equipped with TB diagnostic capabilities and undergoing relevant tests shortly after their initial care-seeking.

Unlike previous studies that focused solely on patients diagnosed using rapid diagnostic tests,^10,11^ our study included patients diagnosed using either rapid or phenotypic methods, allowing a more comprehensive evaluation of the delays. We discovered that the most challenging part of diagnosing DR-TB lay in the detection of RIF resistance compared with patient delay and TB diagnostic delay. In the study, referring to proper hospitals and initiating a DS-TB regimen with ongoing DST were significantly associated with shorter RR-TB diagnostic delays. These results reiterated the call for simultaneous DST assessments in all patients suspected of active TB. From a clinical perspective, it is reasonable to initiate the treatment for DS-TB before the results of DST are available. However, these results should be closely monitored to confirm resistance profiles, and the treatment regimen should be adjusted promptly. This is the main difference between the first and second delay modes in the study. Patients in Mode 1 did not subsequently complete DST when initiating treatment for DS-TB, thereby missing the opportunity to identify drug resistance. Additionally, given the 7.4% prevalence of isoniazid resistance among treatment-naïve patients, rapid testing for resistance to other drugs—such as ethambutol, fluoroquinolones, and isoniazid—is also crucial.^20^

The median treatment delay of RR-TB patients in the study was 10 days, which was generally consistent with previous studies.^11,21^ This study found that rapid molecular testing accelerated the initiation of standard treatment for these patients, which aligned with the findings from a systematic review.^22^ We found that those patients initiating DS-TB regimen with ongoing DST had a significantly higher risk of treatment delay, possibly because the hospitals these patients diagnosed with RR-TB had the capability to diagnose RR-TB but lacked the capacity to treat RR-TB. As a result, these patients required a referral process to access RR-TB treatment.

Interestingly, we found that the total median time from the onset of illness to the initiation of treatment was significantly longer than the sum of the median times for each cascade stage. This suggested the heterogeneity in delays among patients. Every patient without delay was similar, but each patient experiencing a diagnosis or treatment delay faced unique challenges in accessing care. These findings suggested that accelerating the diagnosis and treatment of TB required the coordinated efforts of multiple cascade stages and stakeholders.

Our study had some notable limitations. First, the sample size was relatively small, and it was challenging to avoid recall bias in this study due to the inherent limitations of retrospective studies. Second, the study did not collect the resistance patterns of isolates from possible index cases, which was an important factor affecting the occurrence of diagnostic delay.^23^ Third, we did not follow up on the patient’s final treatment outcomes, which could be analysed further. Fourth, it is likely that we missed patients who never sought healthcare and therefore remained undiagnosed; this suggests that our study cohort may reflect comparatively less severe diagnostic and treatment delays among those with RR-TB.

## CONCLUSIONS

Patients with RR-TB experienced complicated diagnostic and treatment pathways, and the delay between TB diagnosis and RR-TB was the longest. Raising awareness of DST among doctors and patients and improving the availability of DST are urgently needed.

## Supplementary Material


