# Supplementary material for: Patient pathway analysis of rifampicin-resistant TB diagnostic and treatment delays
Source: IJTLD Open. 2025 Feb 1;2(2):90–5. doi: 10.5588/ijtldopen.24.0469 (PMC11827674; doi:10.5588/ijtldopen.24.0469)
Supplement: Supplementary file 1 [file ijtldopen24-0469_supplementarydata1.docx]

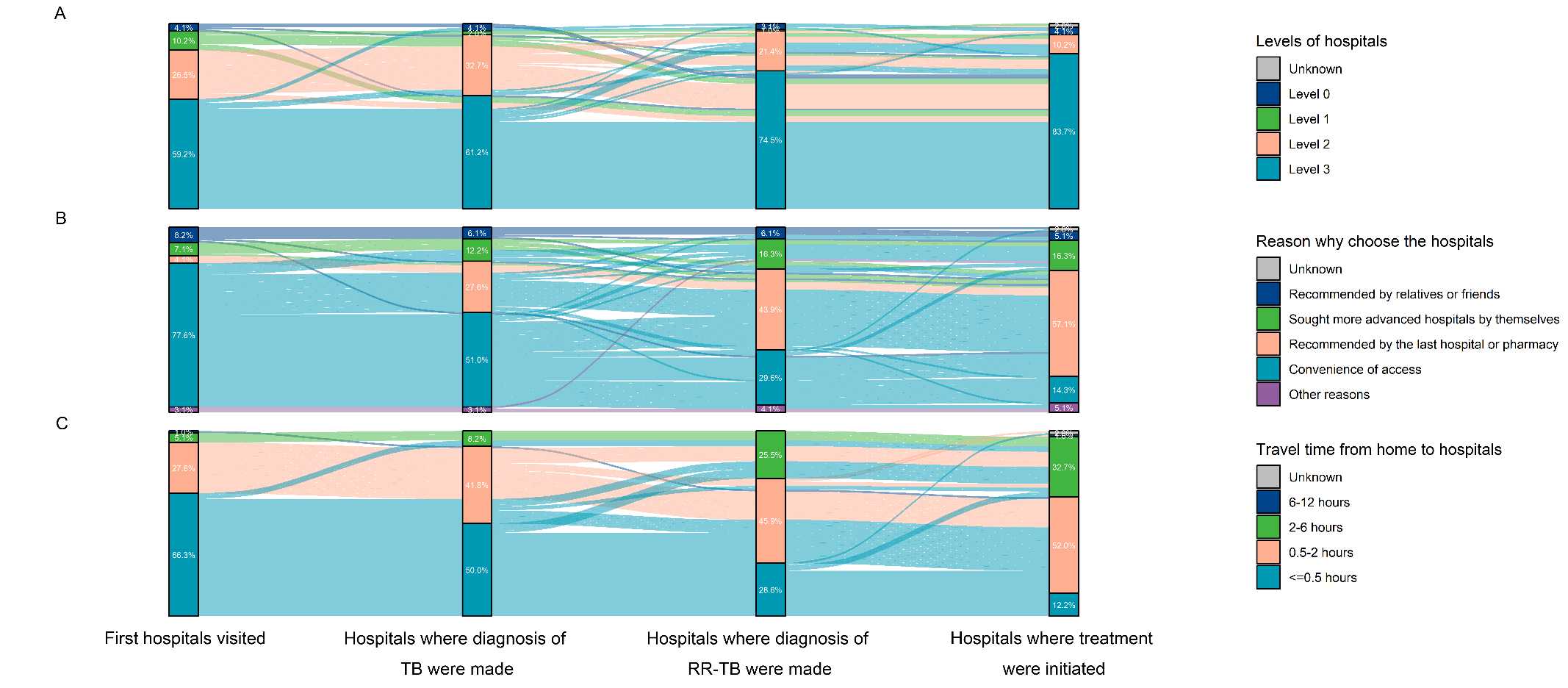


Figure S1. The hospital level (A), the reason why participants chose the hospital (B) and the travel time from home to the hospitals (C) of key hospitals of the participants. Abbreviations: TB: tuberculosis; RR-TB: rifampicin-resistant tuberculosis;

**Table S1. Classification of hospitals based on the three-tier hospital system**

| Hospital Level | Hospital Type | Beds | Equipment | Staffs |
| --- | --- | --- | --- | --- |
| Level 0 | Private clinics or community health sectors | Not available | Not available | 2–3 clinicians trained for basic medical care |
| Level 1 | Hospitals among several communities | 20–99 | Basic clinical departments including laboratories, X-ray room, etc. | 5–10 clinicians and several paramedical staffs |
| Level 2 | Hospitals among a district or town | 100–499 | A diversity of clinical and auxiliary departments. | 150–800 clinicians and paramedical staffs |
| Level 3 | Hospitals among a city or province | >500 | Comprehensive clinical departments as well as the most advanced laboratories and radiologic rooms | >1000 clinicians and paramedical staffs |

**Table S2. Baseline characteristics of study population by hospital^*^**

| **Variable** | **Hospital 1**,  n = 19 | **Hospital 2**, n = 5 | **Hospital 3**, n = 10 | **Hospital 4**, n = 20 | **Hospital 5**, n = 4 | **Hospital 6**, n = 4 | **Hospital 7**, n = 28 | **Hospital 8**,  n = 8 | **p-value** |
| --- | --- | --- | --- | --- | --- | --- | --- | --- | --- |
| No. of patients eligible, n | 23 | 5 | 10 | 28 | 4 | 4 | 46 | 9 | / |
| No. of patients included, n (%^#^) | 19 (82.6%) | 5 (100.0%) | 10 (100.0%) | 20 (71.4%) | 4 (100.0%) | 4 (100.0%) | 28 (60.9%) | 8 (88.9%) | 0.061 |
| Age (year), median (IQR) | 42 (25, 54) | 42 (26, 54) | 41 (28, 49) | 37 (28, 50) | 34 (27, 40) | 69 (62, 74) | 49 (37, 57) | 31 (28, 55) | 0.076 |
| Female, n (%) | 4 (21.1%) | 1 (20.0%) | 3 (30.0%) | 11(55.0%) | 3 (75.0%) | 1 (25.0%) | 7 (25.0%) | 4 (50.0%) | 0.148 |
| Living in rural, n (%) | 16 (84.2%) | 4 (80.0%) | 8 (80.0%) | 7 (35.0%) | 1 (25.0%) | 4 (100.0%) | 15 (53.6%) | 1 (12.5%) | <0.001 |
| Annual household income per capita (US$), Median (IQR) | 1644  (1096, 4932) | 4110  (2384, 5342) | 1370  (1164, 2568) | 3356  (2260, 4933) | 4658  (2740, 6644) | 1781  (616, 4794) | 4110  (2055, 6849) | 7123  (3938, 12055) | 0.025 |
| Smoking history, n (%) | 10 (52.6%) | 3 (60.0%) | 3 (30.0%) | 5 (25.0%) | 2 (50.0%) | 2 (50.0%) | 13 (46.4%) | 2 (25.0%) | 0.544 |
| Alcohol consumption, n (%) | 2 (10.5%) | 1 (20.0%) | 0 (0.0%) | 1 (5.0%) | 0 (0.0%) | 2 (50.0%) | 7 (25.0%) | 0 (0.0%) | 0.098 |
| Previous history of TB, n (%) | 7 (36.8%) | 3 (60.0%) | 2 (20.0%) | 6 (30.0%) | 2 (50.0%) | 1 (25.0%) | 9 (32.1%) | 5 (62.5%) | 0.570 |
| Diabetes mellitus, n (%) | 4 (21.1%) | 0 (0.0%) | 1 (10.0%) | 2 (10.0%) | 1 (25.0%) | 1 (25.0%) | 8 (28.6%) | 0 (0.0%) | 0.464 |
| BMI less than 18.5, n (%) | 9 (47.4%) | 1 (20.0%) | 6 (60.0%) | 3 (15.0%) | 1 (25.0%) | 1 (25.0%) | 4 (14.3%) | 3 (37.5%) | 0.049 |
| Onset of illness, n (%) |  |  |  |  |  |  |  |  |  |
| Any cough | 16 (84.2%) | 2 (40.0%) | 7 (70.0%) | 13 (65.0%) | 3 (75.0%) | 3 (75.0%) | 19 (67.9%) | 5 (62.5%) | 0.703 |
| Sputum | 10 (52.6%) | 0 (0.0%) | 6 (60.0%) | 10 (50.0%) | 3 (75.0%) | 1 (25.0%) | 13 (46.4%) | 3 (37.5%) | 0.375 |
| Weak | 11 (57.9%) | 0 (0.0%) | 5 (50.0%) | 7 (35.0%) | 3 (75.0%) | 1 (25.0%) | 9 (32.1%) | 3 (37.5%) | 0.216 |
| Fever | 7 (36.8%) | 1 (20.0%) | 4 (40.0%) | 8 (40.0%) | 0 (0.0%) | 1 (25.0%) | 8 (28.6%) | 3 (37.5%) | 0.882 |
| Prolonged cough (≥2 weeks) | 8 (42.1%) | 1 (20.0%) | 2 (20.0%) | 4 (20.0%) | 0 (0.0%) | 1 (25.0%) | 7 (25.0%) | 4 (50.0%) | 0.531 |
| Radiographic abnormalities | 1 (5.3%) | 1 (20.0%) | 1 (10.0%) | 3 (15.0%) | 1 (25.0%) | 1 (25.0%) | 5 (17.9%) | 1 (12.5%) | 0.786 |
| Previous DS-TB treatment, n (%) | 17 (89.5%) | 3 (60.0%) | 6 (60.0%) | 14 (70.0%) | 2 (50.0%) | 4 (100.0%) | 19 (67.9%) | 3 (37.5%) | 0.128 |
| Duration of symptoms at presentation, median (IQR) | 39 (12, 187) | 262  (136, 382) | 31 (0, 124) | 17 (6, 61) | 11 (8, 13) | 169  (110, 182) | 30 (12, 168) | 21 (6, 30) | 0.626 |

Abbreviations: IQR: interquartile range; BMI: body mass index; TB: tuberculosis

^*^Hospitals 1–8 represent Affiliated Hospital of Zunyi Medical University (Hospital 1), the Third People's Hospital of Bijie (Hospital 2), the Third People's Hospital of Liupanshui (Hospital 3), Guiyang Public Health Clinical Centre (Hospital 4), the People's Hospital of Anshun (Hospital 5), the First People's Hospital of Huaihua (Hospital 6), Hunan Province Chest Hospital (Hospital 7), and Hangzhou Red Cross Hospital (Hospital 8), respectively.

^#^The percentage is calculated as (Number of Participants included in the study / Numbers of participants eligible in the hospital) × 100%.

**Table S3. Diagnostic test coverage and utilization by visit number (1–5 visits)**

| Visits | Percentage type | Radiography | Smear | Culture | Phenotypic DST | Molecular DST |
| --- | --- | --- | --- | --- | --- | --- |
| 1 | Service coverage rate | 97.8% (90/92) | 71.7% (66/92) | 68.5% (63/92) | 40.7% (37/91) | 40.2% (37/92) |
|  | Service utilization rate | 92.2% (83/90) | 68.2% (45/66) | 49.2% (31/63) | 13.5% (5/37) | 48.6% (18/37) |
| 2 | Service coverage rate | 97.4% (76/78) | 93.6% (73/78) | 92.3% (72/78) | 70.5% (55/78) | 67.9% (53/78) |
|  | Service utilization rate | 69.7% (53/76) | 74.0% (54/73) | 61.1% (44/72) | 10.9% (6/55) | 56.6% (30/53) |
| 3 | Service coverage rate | 100.0% (46/46) | 93.5% (43/46) | 93.5% (43/46) | 71.7% (33/46) | 67.4% (31/46) |
|  | Service utilization rate | 65.2% (30/46) | 74.4% (32/43) | 62.8% (27/43) | 24.2% (8/33) | 64.5% (20/31) |
| 4 | Service coverage rate | 100.0% (30/30) | 96.7% (29/30) | 96.7% (29/30) | 70.0% (21/30) | 66.7% (20/30) |
|  | Service utilization rate | 63.3% (19/30) | 72.4% (21/29) | 58.6% (17/29) | 23.8% (5/21) | 50.0% (10/20) |
| 5 | Service coverage rate | 100.0% (21/21) | 100.0% (21/21) | 100.0% (21/21) | 71.4% (15/21) | 66.7% (14/21) |
|  | Service utilization rate | 52.4% (11/21) | 66.7% (14/21) | 57.1% (12/21) | 6.7% (1/15) | 35.7% (5/14) |

Service coverage rate = the number of hospitals that had the capability to offer the specific service / the number of hospitals that were surveyed during each visit

Service utilization rate = the number of patients who performed the specific test / the number of hospitals that had the capability to offer the test during each visit

**Table S4 The diagnosis and treatment delay characteristics of the eight participants who couldn’t be summed into the three modes***

|  | Initially DS-TB, n = 7 | Loss to follow up after TB diagnosis, n = 1 |  |
| --- | --- | --- | --- |
| Delay (days), median (IQR) | | | |
| From onset to first visit | 14.0 (10.0, 92.5) | 2.0 |  |
| From first visit to TB diagnosis | 4.0 (0.0, 23.5) | 0.0 |  |
| From TB diagnosis to RR-TB diagnosis | 210.0 (175.0, 898.5) | 32.0 |  |
| From RR-TB diagnosis to RR-TB treatment | 9.0 (5.0, 29.5) | 1,132.0 |  |
| Duration of treatment for DS-TB (days), median (IQR) | 164.0 (109.5, 275.5) | - |  |

* Seven were initially identified of DS-TB and were diagnosed with RR-TB later, while one received phenotypic DST at TB diagnosis but did not initiate any anti-TB treatment for economic reason.

Abbreviations: IQR: interquartile range; TB: tuberculosis; RR-TB: rifampicin-resistant tuberculosis; DS-TB: drug-susceptible tuberculosis.

**Table S5 Characteristics of all variables associated with RR-TB diagnosis and RR-TB treatment.**

|  | n (%) | Univariable Cox regression, HR (95% CI, p) | |
| --- | --- | --- | --- |
|  |  | RR-TB diagnosis | RR-TB treatment initiation |
| Age | - | 1.00 (0.99–1.02, p=.551) | 1.02 (1.01–1.03, p=.002) |
| Male | 64 (65.3%) | 0.87 (0.57–1.32, p=.509) | 0.79 (0.52–1.22, p=.290) |
| Farmers | 21 (21.4%) | 1.12 (0.68–1.84, p=.666) | 1.33 (0.81–2.16, p=.257) |
| Living in urban area | 42 (42.9%) | 1.40 (0.93–2.09, p=.105) | 1.09 (0.72–1.64, p=.690) |
| Unmarried | 28 (28.6%) | 0.78 (0.50–1.23, p=.287) | 0.68 (0.43–1.08, p=.105) |
| High school Degree or above | 38 (38.8%) | 1.43 (0.95–2.16, p=.087) | 0.98 (0.65–1.50, p=.936) |
| Had health insurance | 96 (98.0%) | 0.89 (0.22–3.62, p=.868) | 0.77 (0.19–3.16, p=.722) |
| Annual household income per capita (USD)* | - | 1.00 (1.00–1.00, p=.961) | 1.00 (1.00–1.00, p=.900) |
| Smoking history | 40 (40.8%) | 0.99 (0.66–1.48, p=.956) | 0.64 (0.42–0.96, p=.033) |
| Alcohol consumption | 13 (13.3%) | 1.36 (0.76–2.46, p=.302) | 1.30 (0.72–2.35, p=.379) |
| TB history | 35 (35.7%) | 0.82 (0.54–1.25, p=.362) | 1.16 (0.76–1.76, p=.504) |
| Diabetes | 17 (17.3%) | 1.35 (0.79–2.31, p=.271) | 1.41 (0.83–2.41, p=.203) |
| Chronic bronchitis | 11 (11.2%) | 0.85 (0.44–1.65, p=.632) | 1.23 (0.65–2.32, p=.518) |
| Chronic cough | 12 (12.2%) | 0.72 (0.38–1.35, p=.302) | 1.25 (0.68–2.31, p=.476) |
| Silicosis | 2 (2.0%) | 1.66 (0.41–6.80, p=.479) | 0.53 (0.13–2.18, p=.381) |
| BMI less than 18.5 | 28 (28.6%) | 0.92 (0.59–1.43, p=.711) | 0.94 (0.60–1.46, p=.771) |
| Had sputum before first visit | 46 (46.9%) | 1.29 (0.87–1.93, p=.209) | 0.68 (0.45–1.02, p=.065) |
| Hemoptysis before first visit | 16 (16.3%) | 1.12 (0.65–1.91, p=.692) | 0.72 (0.41–1.25, p=.240) |
| Had prolonged cough (>=2 weeks) before first visit | 27 (27.6%) | 0.66 (0.42–1.04, p=.076) | 1.09 (0.69–1.72, p=.698) |
| Had no symptom before first visit | 14 (14.3%) | 1.19 (0.67–2.10, p=.560) | 0.74 (0.42–1.31, p=.303) |
| Understand the curability of tuberculosis | 72 (73.5%) | 0.82 (0.52–1.30, p=.399) | 0.94 (0.60–1.48, p=.785) |
| Had Stigma regarding TB | 49 (50.0%) | 0.52 (0.34–0.80, p=.002) | 1.36 (0.90–2.04, p=.140) |
| Received DS-TB regimens before RR-TB diagnosis | 68 (69.4%) | 0.07 (0.04–0.15, p<.001) | 0.89 (0.57–1.38, p=.608) |
| First diagnosed by molecular DST (vs. by phenotypic DST) | 80 (81.6%) | 1.29 (0.77–2.17, p=.337) | 2.41 (1.37–4.23, p=.002) |
| Proper hospitals were recommended at TB diagnosis | 35 (35.7%) | 1.70 (1.12–2.60, p=.014) | 0.85 (0.55–1.29, p=.440) |
| The hospital level of the first hospital visited |  |  |  |
| Level 0 or 1 | 6 (6.1%) | Reference | Reference |
| Level 2 | 32 (32.7%) | 0.98 (0.41–2.36, p=.968) | 1.03 (0.40–2.65, p=.956) |
| Level 3 | 60 (61.2%) | 1.06 (0.46–2.46, p=.894) | 0.97 (0.39–2.44, p=.954) |
| Proper hospitals were recommended when diagnosed with RR-TB | 26 (26.5%) | -^#^ | 0.92 (0.58–1.46, p=.713) |
| Diagnosing TB at TB designated hospitals^*^ | 75 (81.5%) | 1.28 (0.73–2.26, p=.384) | 0.78 (0.46–1.34, p=.375) |
| Molecular DST available at TB diagnosis* | 61 (62.9%) | 1.52 (1.00–2.32, p=.052) | 1.08 (0.71–1.66, p=.717) |
| Number of hospitals visited before RR diagnosis | - | 0.68 (0.52–0.88, p=.003) | 0.76 (0.60–0.96, p=.021) |
| Delay mode^&^ |  |  |  |
| Mode 1 | 31 (31.6%) | Reference | Reference |
| Mode 2 | 30 (30.6%) | 2.89 (1.70–4.90, p<.001) | 0.63 (0.38–1.06, p=.082) |
| Mode 3 | 29 (29.6%) | 27.01 (11.95–61.05, p<.001) | 1.01 (0.59–1.70, p=.980) |
| Others | 8 (8.2%) | 0.64 (0.28–1.46, p=.288) | 0.56 (0.25–1.24, p=.155) |

^*^There were missing values.

^#^This variable was not considered as the potential factor associated with RR-TB diagnosis.

^&^Mode 1 refers to those who initiated DS-TB treatment without concern for drug resistance; Mode 2 refers to those who initiated DS-TB treatment with ongoing DST; Mode 3 refers to those who received molecular DST at TB diagnosis.

Abbreviations: RR-TB: rifampicin-resistant tuberculosis; HR: hazard ratio; CI: confidence interval; TB: tuberculosis; BMI: body mass index; DS-TB: drug-susceptible tuberculosis; DST: drug susceptibility testing;

**Table S6 Risk factors associated with RR-TB treatment initiation**

|  | Univariable Cox regression | Multivariable Cox regression |
| --- | --- | --- |
|  | HR (95% CI, p) | Adjusted HR (95% CI, p) |
| Age | 1.02 (1.01–1.03, p=.002) | 1.03 (1.01–1.04, p<.001) |
| Smoking history | 0.64 (0.42–0.96, p=.033) | 0.68 (0.42–1.11, p=.122) |
| Having sputum before first visit | 0.68 (0.45–1.02, p=.065) | 0.77 (0.51–1.17, p=.217) |
| Diagnosed by molecular DST with RR-TB (vs. by phenotypic DST) | 2.41 (1.37–4.23, p=.002) | 2.87 (1.45–5.65, p=.002) |
| Number of hospitals visited before RR diagnosis | 0.76 (0.60–0.96, p=.021) | 0.78 (0.58–1.06, p=.117) |
| Delay mode^*^ |  |  |
| Mode 1 | Reference | Reference |
| Mode 2 | 0.63 (0.38–1.06, p=.082) | 0.52 (0.28–0.96, p=.038) |
| Mode 3 | 1.01 (0.59–1.70, p=.980) | 0.72 (0.35–1.49, p=.375) |
| Others | 0.56 (0.25–1.24, p=.155) | 0.66 (0.26–1.68, p=.386) |

^*^Mode 1 refers to those who initiated DS-TB treatment without concern for drug resistance; Mode 2 refers to those who initiated DS-TB treatment with ongoing DST; Mode 3 refers to those who received molecular DST at TB diagnosis.

Abbreviations: RR-TB: rifampicin-resistant tuberculosis; HR: hazard ratio; CI: confidence interval; DST: drug susceptibility test
